# Staring at the Naked Goddess: Unraveling the Structure and Reactivity of Artemis Endonuclease Interacting with a DNA Double Strand

**DOI:** 10.3390/molecules26133986

**Published:** 2021-06-29

**Authors:** Cécilia Hognon, Antonio Monari

**Affiliations:** LPCT, Université de Lorraine and CNRS, UMR 7019 LPCT, F-54000 Nancy, France; cecilia.hognon@univ-lorraine.fr

**Keywords:** Artemis endonuclease, DNA lesion repair, classical molecular dynamics, quantum mechanics/molecular mechanics, reaction free energy profiles

## Abstract

Artemis is an endonuclease responsible for breaking hairpin DNA strands during immune system adaptation and maturation as well as the processing of potentially toxic DNA lesions. Thus, Artemis may be an important target in the development of anticancer therapy, both for the sensitization of radiotherapy and for immunotherapy. Despite its importance, its structure has been resolved only recently, and important questions concerning the arrangement of its active center, the interaction with the DNA substrate, and the catalytic mechanism remain unanswered. In this contribution, by performing extensive molecular dynamic simulations, both classically and at the hybrid quantum mechanics/molecular mechanics level, we evidenced the stable interaction modes of Artemis with a model DNA strand. We also analyzed the catalytic cycle providing the free energy profile and key transition states for the DNA cleavage reaction.

## 1. Introduction

Artemis is an endonuclease [1,2,3] that plays a fundamental role in processing DNA strands in immune system cells, allowing their recombination and hence the maturation of the immune response. Indeed, Artemis is present in B and T cells, and its catalytic activity concerns the opening of the DNA hairpins in the V(D)J process. V(D)J [4,5] is the mechanism through which immune system cells assemble variable (V), diversity (D), and joining (J) gene sequences responsible for the production of immunoglobulins (Igs) and T cell receptors that can recognize a large number of antigens. Thus, Artemis is a key component allowing the somatic development of Igs and T cells in eukaryotes. Its activity is also finely regulated by specific cellular pathways, and namely Artemis forms a complex with the DNA-dependent protein kinase catalytic subunit (DNA-Pkcs), which induces phosphorylation and activation of the enzyme, most probably by exposing its catalytic site [6,7]. Thus, the activation of Artemis allows the opening of the DNA hairpins that have been generated by the recombination activated gene during V(D)J [8]. Importantly, since the Artemis/DNA-Pkcs is the only protein complex able to effectively open DNA hairpins [9], functional inhibiting mutations in either partner effectively block B and T cell maturation and lead to phenotypes showing severe combined immunodeficiency [10] and to the accumulation of hairpin coding ends in thymocytes [9]. In addition to its fundamental role in immune system maturation, Artemis has also been associated with the capacity for processing DNA lesions, and in particular strand breaks, thus participating in maintaining genome stability [11,12,13]. Indeed, non-functional mutations of Artemis have been correlated with an increased radiosensitivity [10] of B and T cells, suggesting its participation to the strand break repair machinery [14]. Notably, it has been shown in cellular models that Artemis is recruited to the site of DNA damage and acts in a way that is similar to non-homologous end-joining pathways to assure repair [15,16]. Interestingly, the interplay between DNA repair and immune system maturation has also been highlighted, in the sense that DNA repair machinery can participate in the last steps of the V(D)J process [10].

Artemis shows sequence similarity and homology with a number of RNA ribonucleases and two human nucleases of the SNM1 family: SNM1A and SNM1B/Apollo; hence, it belongs to the metallo-β-lactamase class [6]. Despite obvious similarities, Apollo, the twin counterpart of Artemis, presents only an exonuclease activity, and from a structural point of view it presents a reduced density of positively charged amino acids [17].

As a matter of fact, the function and the biological role of Artemis could potentially result in a most ideal target candidate for chemotherapy or radiotherapy sensitization [18,19]. Furthermore, its participation in immune system maturation could also point to the possibility of exploiting its action in immunotherapy strategies. However, the structure of the catalytic and DNA binding domains of Artemis has been resolved only very recently. Furthermore, while the protein was correctly resolved, no bound nucleic acid was shown in the obtained crystallographic structure [20]. The lack of a precise structural and dynamic characterization of Artemis’s behavior clearly hampers the rational development of potential inhibitors that could be used as chemotherapeutics or as radiotherapy sensitizers.

From a biophysical point of view, different structural characteristics of Artemis have been pinpointed, notably by Karim et al. [20]. These include the presence of a zinc finger and a surface groove presenting a high density of positively charged amino acids, mainly lysines, which should be essential for DNA recognition and stabilization. From a chemical point of view, exo- or endonuclease activity is usually performed by a catalytic site featuring a metal or a metal cluster, usually Zn^2+^ or Mg^2+^, which acts through mono or bimetallic pathways [21]. In the case of Artemis, only one Zn^2+^ ion has been located in the active site. However, also considering the similarity with the other members of the SNM1 family that exert bimetallic activity, it seems reasonable that a bimetallic cluster should be present in the biological active form. Furthermore, despite the presence of zinc in the crystal structure, the evidence of sustained activity of the enzyme in a magnesium buffer [20] strongly points towards the possibility of a bimetallic (Mg^2+^)_2_ active center.

In the present contribution, we resort to high-level molecular modeling and simulation to explore the inherent structural properties of Artemis and its interaction with DNA double strands. In addition, we will also examine the reaction profile leading to the catalytic DNA strand break confirming the activity of the (Mg^2+^)_2_ cluster. Note that to maintain coherence with the experimental available crystallographic structure, we have considered a double-stranded DNA fragment instead of a hairpin one. Even if in this case we are missing a key crucial biological partner of Artemis, we are confident that the main aspects of the repair mechanism may indeed be inferred, even if some caution should be considered. To this aim, we consider a multiscale approach combining both full-atom classical molecular dynamics (MD) simulations exceeding the μs timescale to explore the DNA binding and its structural evolution, with a hybrid quantum mechanics/molecular mechanics (QM/MM) approach. The latter involves enhanced sampling strategies, namely umbrella sampling (US), to obtain the free energy profile (FEP) of the most relevant and critical steps of the DNA cleavage. Hence, through our work we provide a unified vision of Artemis functioning, resolving the still elusive or contrasting hypotheses regarding this crucial protein.

## 2. Results

We first performed an MD simulation of the unbound wild-type form of the catalytic domain of human Artemis starting from the structure reported by Karim et al. (pdb: 6wo0) [20]. In order to better describe the bimetallic cluster constituting the catalytic site, we included a second Mg^2+^ ion together with bonding constraints on the two metals to avoid instability due to positive charge repulsion. As shown in SI, and as witnessed by the small value of the root-mean-square deviation (RMSD) all along the trajectory, the protein structure was stable, with deviations plateauing at 3.8 ± 0.6 Å. Most importantly, the main secondary and tertiary structural motifs, in particular two extended β-sheet regions connected by loops and α-helices, were preserved all along the dynamic. A positively charged groove, composed mainly of lysine and approaching the catalytic active site, was also evident and solvent exposed, constituting an optimal target for the DNA binding. Interestingly, along the MD simulation of the isolated protein, the charged groove also showed a remarkable stability with only limited oscillation of its width and depth, an occurrence that could be ideal for allowing efficient binding with the nucleic acid.

**Figure 1:**
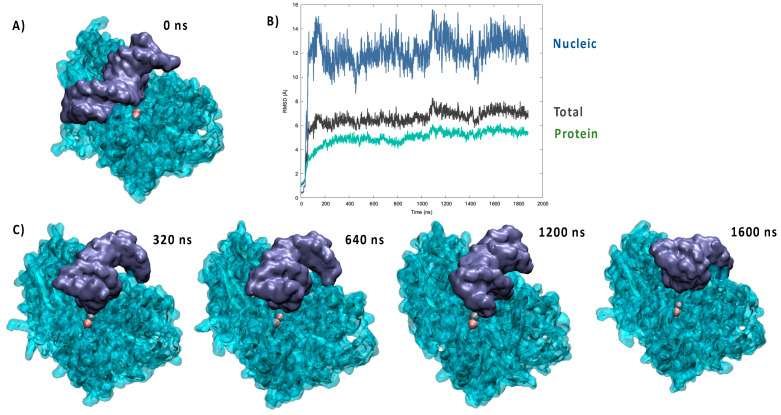
(**A**) Most favorable pose resulting from the docking of the crystal structure of Artemis with a DNA double strand. (**B**) Time evolution of the RMSD for the MD simulation of the Artemis/DNA complex. (**C**) Representative snapshots extracted at different time frames of the MD simulation.

In the crystal structure of pdb: 6wo0 the bound DNA strand was not resolved. Hence, we first performed a protein/DNA docking analysis to identify suitable starting poses. As shown in Figure 1A, and unsurprisingly, the most favorable pose placed the DNA backbone in the positively charged groove of the protein. Although electrostatic interactions appeared evident and favorable to drive the binding, it was also clear that the ideal straight arrangement of the DNA strand used for the docking was not able to maximize the contacts and lead to a compact complex. These general observations were indeed confirmed by the analysis of the MD simulations performed from the docking pose, which points to the establishment of a persistent complex remaining stable for the entire simulation reaching 2 μs. Notably, an independent replica, also spanning 2 μs, was performed, giving similar results (see Supplementary Materials). As shown in Figure 1B, the time evolution of the RMSD for the global Artemis/DNA complex remained globally moderate and was stabilized at around 6.5 ± 0.9 Å. On the contrary, the contribution due to the nucleic acid was clearly more important, and while averaging at 11.9 ± 2.0 Å, it also showed more pronounced oscillations, which are indicative of a greater flexibility and the possible coexistence of slightly different arrangements.

A part from the analysis of the RMSD, the reorganization of the bound DNA strand was also clearly evidenced by the visual analysis of the trajectory, as shown by the snapshots reported in Figure 1C. Indeed, it is evident that the strand tends to bend considerably to accommodate the protein positively charged groove and maximize the contact. This fact was also evidenced by the analysis of the DNA structural parameters, and notably the global bending, performed with Curves+ and reported in the Supplementary Materials. Interestingly, the bound nucleic acid also tended to show sliding and rotation in the groove, which while maintaining a stable complex may also allow a certain flexibility and the possibility to present different phosphate units to the vicinity of the catalytic site. This aspect can also be related to the biological role of Artemis, which requires the ability to cleave unspecific DNA sequences for repair of V(D)J maturation.

**Figure 2:**
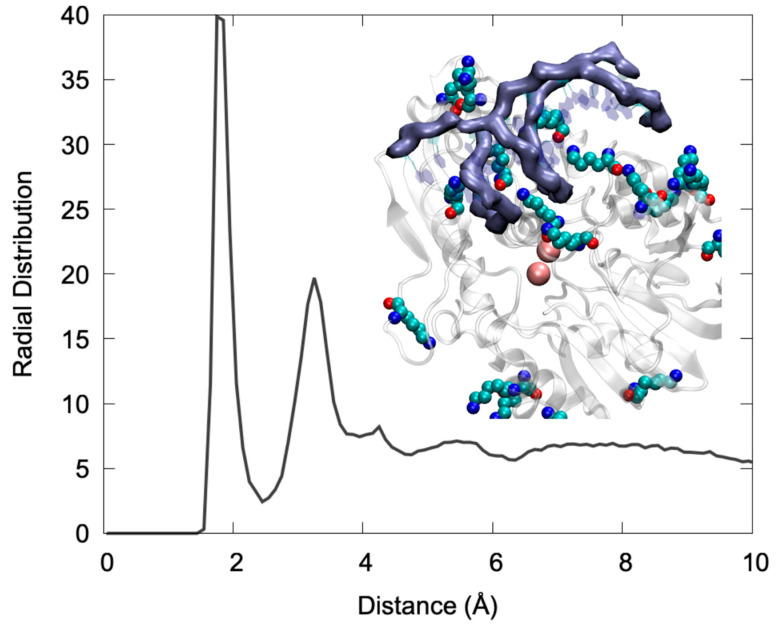
Radial distribution function between the negative O1P and O2P atoms of the DNA backbone and the HZ hydrogen of the LYS ammonium group. A snapshot showing the interaction of LYS charged moieties (in van der Waals representation) with the DNA backbone (highlighted with the purple surface) also shown in the inlay.

Looking more in detail to the specific interactions stabilizing the DNA/Artemis complex (Figure 2), one may see that the binding is mainly driven by an extended salt bridge involving the DNA backbone phosphate and the lysine populating the positively charged groove. This can be clearly appreciated by examining the radial distribution function of the distance between the negatively charged oxygens of the phosphate groups and the hydrogen atom of the positive ammonium moiety in Lys. As shown in Figure 2, the distribution presented a very intense and sharp peak at 2 Å that is indicative of the formation of rather rigid and persistent interactions. Note that a secondary peak was also observed at larger distances, which can be safely attributed to the other ammonium hydrogens. In the snapshot reported in Figure 2, one can also clearly identify that the Lys lateral chains were indeed interacting strongly with both the DNA major and minor groove, thus providing strong anchoring points. Interestingly, the global architecture, involving the penetration of Lys in the DNA grooves, is also compatible with a global combined rotation and translation of the nucleic acid that is reminiscent of a “corkscrew” action. This movement was observed in one of the replica MD simulations (see the Video S1 in Supplementary Materials), and clearly allows the exposure of different phosphate and glycosidic bonds to the vicinity of the active site while preserving the global stability of the complex and the favorable interactions. Once again, this observation is compatible with the recognized capacity of Artemis to process different DNA sequences, notably to allow for V(D)J maturation as well as its recognized exonuclease activity that adds up to the more conventional endonuclease capacity [22].

**Figure 3:**
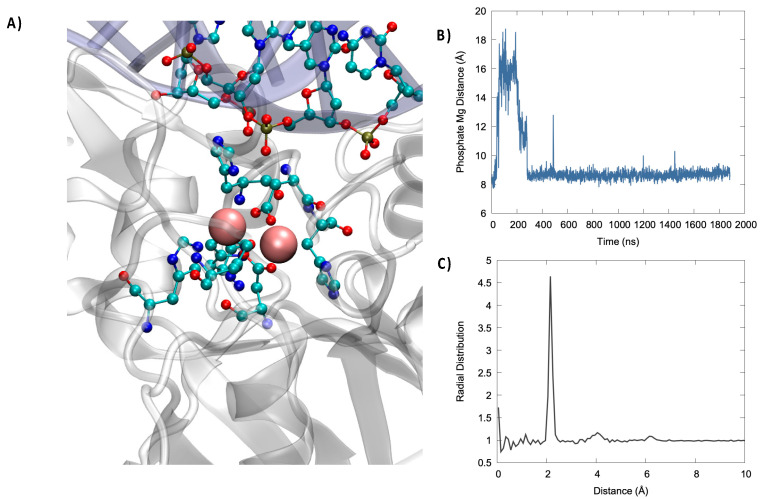
(**A**) Representative snapshot highlighting the catalytic active site organization. (Mg^2+^)_2_ is represented in van der Walls, while the protein amino acids complexing the bimetallic cluster and closest nucleic acid residues are shown in ball and sticks. (**B**) Time series of the evolution of the distance between the phosphate OP atom and the closest Mg^2+^ ion. (**C**) Radial distribution function for the distance between Mg^2+^ and water oxygen atoms.

In Figure 3, we may evidence the main characteristic of the active site of Artemis. Notably, and in addition to the two Mg^2+^ ions, we can also identify negative, or electron-rich, ligands that are necessary to stabilize the metal cluster. In particular, a high density of histidine was present that was susceptible to interact with the magnesium cation. More importantly, the presence of two aspartates, ASP 61 and ASP 160, seems crucial since the harder oxygen may be a better ligand for magnesium than nitrogen. The analysis of the active site, and in particular the lack of an additional Asp ligand compared to other nuclease such as SNM1B/Apollo, also justifies the more labile nature of the second Mg^2+^, which makes it difficult to resolve its position by crystallography. Importantly, the active site was also relatively solvent exposed. As a matter of fact, water molecules interacted strongly with the (Mg^2+^)_2_ cluster, as seen by the sharp peak present in the radial distribution function at 2 Å (Figure 3) and by the fact that, on average, five water molecules were in a radius of 3 Å from Mg^2+^, as can be inferred from the integral of the radial distribution function. In the same context, it is worth mentioning that using classical MD we were only exploring pre-reactive conformation, in which the phosphate group is relatively far from (Mg^2+^)_2_. However, we may observe, as seen in Figure 3, an important stabilization of its distance. Note that in some instances of the second replica, a switch of the closest phosphate oxygen has been observed, coherent with the global motion of the DNA strand described previously. Furthermore, Asp61 and His62 may also have a more stringent function than the simple stabilization of the bimetallic cluster and may instead play an active role in the activation of the water molecule attacking the phosphate bond by acting as a hydrogen acceptor. This is also confirmed by the fact that the mutation of Asp 61 totally suppressed the catalytic activity.

**Figure 4:**
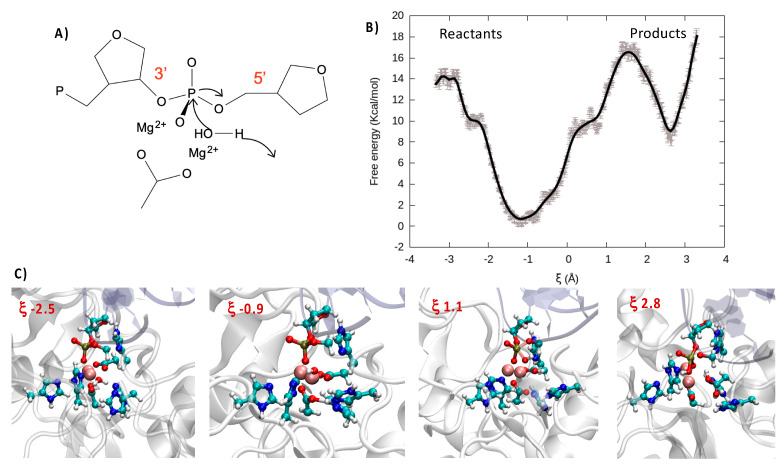
(**A**) Schematic representation of the catalytic attack of a water molecule to the phosphate. (**B**) FEP over the reaction coordinate obtained at DFT level of theory. (**C**) Representative snapshots along the reaction coordinate illustrating the reactant, transition state, and product region. Note that the QM partition is represented in balls and sticks.

Indeed, the catalytic activity of bimetallic endonucleases has been shown to proceed through a relatively straightforward mechanism in which one water molecule, complexed to the metallic cluster, attacks the phosphorus, inducing the breaking of one P-O and thus cleaving DNA (Figure 4A). Importantly, nuclease selectively takes place with a 3′→5′ directionality, thus leading to the production of a P-OH and a sugar-O^−^ fragment, respectively. Moreover, the cleavage of the oxygen–phosphorus bond is also favored by the interaction of the phosphate group with the bimetallic cluster, which facilitates the nucleophilic attack of water. The deprotonation and activation of the nucleophilic moiety is also fundamental in favoring the reaction, hence strengthening the importance of proton/acceptor amino acids in the nearby active site.

In the case of Artemis, the exact nature of the catalytic process is still quite debated, and in particular the capacity of a magnesium cluster for inducing the catalytic reaction and the nature of the deprotonating amino acids are still controversial [20]. For this reason, and having accessed a reasonable structure for the Artemis/DNA complex, we also performed QM/MM enhanced sampling to describe the reaction. Note that in agreement with the most accepted kinetic model for endonucleases, prior to performing the QM/MM simulations, we have forced the DNA phosphate oxygen to reach a distance of only about 3 Å with the (Mg^2+^)_2_ cluster through steered molecular dynamic simulations. For our enhanced sampling simulation, we have considered a reaction coordinate ξ that is defined as the difference between the distance from one hydrogen of the reactive water molecule to the center of mass of the oxygen and nitrogen in the lateral chains of Asp 61 and His 62 and the distance from the oxygen atom of the same water molecule to the reactive phosphorus (see Supplementary Materials for illustration). Thus, while negative values of ξ corresponded to the reactant region, positive values indicated the products. As shown in Figure 4B through the FEP and pictorially in the snapshots of Figure 4C, the initially Mg-bonded water was rapidly deprotonated thanks to the concerted action of Asp 61 and His 62, which participated in the stabilization of the proton. This led to both an enhanced global stabilization of the system, as shown by the shallow minimum at 0.9, and the production of a more nucleophilic species, namely the Mg-bonded OH^−^ anion. Proceeding further along the reaction coordinate, a free energy barrier of about 16 kcal/mol should be overcome to reach the rate-determining transition state (TS) in which the 5′ PO bond is weakened while OH^−^ is approached. As previously observed, the FEP basin appeared quite shallow, and specifically one can observe a plateau at 0.5 (~10.0 kcal/mol), which is the result of the formation of partially stabilizing interactions between OH^-^ and the residues around the catalytic site. The presence of the plateau can also facilitate overcoming the global free energy barrier and thus increase the overall catalytic efficiency. Indeed, once the TS was attended, the FEP evolves sharply and continuously towards a minimum energy region corresponding to the cleaved backbone. In this study we are not taking into account the protonation of the resulting sugar-O^−^ fragment nor the release of the two cleaved DNA fragments that should globally lower the product free energy due to both enthalpic and entropic factors.

## 3. Discussion and Conclusions

The role of endonucleases in general, and Artemis in particular, is essential in assuring both a proper reparation of DNA lesions and the efficient adaptation and maturation of the immune system. This mostly relies on their efficiency and flexibility in cleaving different DNA strands, including hairpins, a flexibility that should find an echo in the peculiar structural and reactive features of the enzyme. Furthermore, the role of Artemis and its importance for T and B cell production also make this protein an attractive target for cancer therapy, including radiotherapy sensitization and immunotherapy. Despite all these considerations, the structure of Artemis has been resolved only recently, and important features such as the precise interaction with DNA or the global structure of the active site have remained elusive and open to question.

In this contribution, thanks to multiscale molecular modeling and simulation, we provide a first rationalization of the interaction between Artemis and DNA. However, caution should be considered since we have only considered a DNA double-strand and not a hairpin structure. More precisely, while providing evidence for the formation of stable complexes with a DNA double-strand, we also clearly identified the Lys-rich groove as the crucial driving force behind the interaction. We have also evidenced that basic amino acids are able to penetrate in the groove of the DNA strand; this in turn provides the possibility of a corkscrew-like movement of the DNA that, while maintaining a tight complex, may lead different phosphate groups to the catalytic site. On the contrary, and despite a slight bending of the strand, no particular structural deformations of the nucleic acid can be highlighted.

From a biochemical point of view, we have shown, thanks to QM/MM US sampling, that the cleavage reaction may proceed through the assistance of a bimetallic (Mg^2+^)_2_ cluster and the crucial participation of the nearby protein residues. In particular, Asp 61 and His 62 were fundamental since they not only acted as ligands for the metals, they also deprotonated the reactive water molecule, thus producing an OH^−^ anion that further attacked the phosphate group. Interestingly enough, the initial deprotonation of water was accompanied by a considerable stabilization of the system, as shown by the FEP. The excess energy released in this step may, in the following, facilitate overcoming the free energy barrier and facilitate the reaction. Interestingly, a relatively small plateau prior to the TS was also evident in the FEP, an occurrence that once again may globally facilitate the catalytic cleavage. Our results, pointing to a Mg-based enzyme, are also coherent with experimental observation such as the activity of Artemis in magnesium buffers. The suppression of the catalytic activity by mutation of Glu 61 can also be explained due to its fundamental role in the first water deprotonation step. Note that our results are coherent with previously admitted endonuclease reaction mechanisms, in particular concerning the participation of a bimetallic cluster [21], and has already cited with biochemical data that point to the loss of activity by mutation of Asp 61 [20].

Our results are important in providing deeper structural and mechanistic insights on the activity of Artemis, and in the future we plan to extend them in providing suggestions for the rational design of suitable inhibitors. In parallel, we also plan to increase the study of the endo and exonuclease Pantheon by providing detailed modeling and simulation of the related SNM1 proteins such as SNM1B/Apollo.

## 4. Materials and Methods

The recently reported [20] crystal structure of Artemis’s catalytic domain was retrieved from the pdb data base (pdb: 6wo0). To obtain an initial structure of the Artemis/DNA complex, an ideal B-DNA double strand, having the same sequence as the one used by Karim et al. [20] for Artemis crystallography (5′-cacagctgatcgc-3′), was built using the nucleic acid builder (nab) utilities of Amber [23]. The protein and the nucleic acid were docked using the NPDock webserver utility [24,25]. All stable poses obtained presented the interaction of the DNA strand in the positively charged groove of the protein and were basically equivalent; hence, only the highest scoring pose was kept for the subsequent molecular simulations.

The docked complex was solvated in a cubic water box with a buffer of 9 Å, and K^+^ cations were added to ensure electroneutrality. Since only metal atoms were present in the catalytic center, the second Mg^2+^ was manually added and its position inferred by superposing the crystal structure of Artemis with the one of Apollo exonuclease (pdb: 5aho), for which a totally resolved active site is provided [17]. The protonation state of the amphipathic residues was determined according to their pKa, estimated with propka [26], with the exception of the His and Cys present in the catalytic site and in a Zn-finger region whose state was assigned to maximize the interactions with the metals. The protein and the nucleic acid were modelled with the amberf99sb force field [27], including the bsc1 corrections for DNA [28,29], while water was modelled using TIP3P [30]. The amino acids composing the Zn-finger domain were described using a recently developed amber-based non-bonded potential [31]. To assure the stability of the bimetallic catalytic site, additional constraints to the Mg-Mg distance as well as to the distance of Mg^2+^ with the nitrogen atom of the first-shell His residues were applied. Two independent replicas were constructed, and MD simulation was performed using the NAMD code [32,33] in the constant temperature (300 K) and pressure (1 atm) thermodynamic ensemble (NPT) enforced using the Langevin thermostat [34] and barometer [35]. After 5000 minimization steps, the system was equilibrated for 9 ns, during which constraints on the heavy atoms were progressively released, and finally a 2.0 μs production run for the first replica and 2.5 μs for the second replica were performed. The use of a hydrogen mass reparation (HMR) strategy [36] in combination with the Rattle and Shake procedure [37] allowed the use of a 4 fs timestep. Particle mesh Ewald (PME) [38] was used to treat long-range interactions with a cut-off of 9 Å.

For the determination of the reaction mechanism, the electrostatic embedding QM/MM strategy [39] was followed, after having preliminarily decreased the phosphate–Mg distance by steered molecular dynamics. The chosen QM partition involved the (Mg^2+^)_2_ cluster; the reactive water molecule; the lateral chains of His57, His59, His62, His139, His343, Asp61, and Asp160; the 3′ phosphate and sugar moiety; and the 5′ OCH_2_ group. Dangling bonds were saturated with the link-atom approach, thus adding hydrogens, leading to a total of 100 QM atoms. After equilibration, the US was performed by partitioning the reaction coordinates in 67 windows spaced by 0.1 Å and spanning the −3.3/3.3 Å domain; each window was preliminary equilibrated for 500 fs. QM calculation was performed at density functional theory (DFT) level using the ωB97X functional [40] and the 6-31G basis set. A time step of 1 fs was consistently used, while Rattle and Shake was removed for the QM partition. Note that dispersion was not taken into account explicitly in DFT to avoid double counting due to the inclusion of van der Waals terms in the QM/MM Hamiltonian. QM/MM calculations were performed using the Amber/Terachem interface [41,42,43]. The FEP was reconstructed using the weighted histogram analysis method (WHAM) algorithm [44,45]. The overlapping between the windows was checked and can be appreciated in SI. Trajectories issued from both classical and QM/MM simulations were visualized and analyzed using VMD [46], and the structural parameters of DNA were obtained using Curves+ [47].

## Supplementary Materials

The following are available online. Figure S1 RMSD for the second replica, Figure S2 representative snapshots of the trajectory, Figure S3 RMSD for the isolated protein, Figure S4 DNA structural parameters obtained with Curves+, Figure S5 representation of the QM partition, Figure S6 representation of the reaction coordinate, Figure S7 overlap between the umbrella sampling windows. Video S1 Trajectory of the MD simulation for the Artemis/DNA simulation: artemis_video.mpg.
