# Supplementary material for: Staring at the Naked Goddess: Unraveling the Structure and Reactivity of Artemis Endonuclease Interacting with a DNA Double Strand
Source: Molecules. 2021 Jun 29;26(13):3986. doi: 10.3390/molecules26133986 (PMC8271620; doi:10.3390/molecules26133986)

checkCIF/PLATON report

Structure factors have been supplied for datablock(s) 1

THIS REPORT IS FOR GUIDANCE ONLY. IF USED AS PART OF A REVIEW PROCEDURE FOR PUBLICATION, IT SHOULD NOT REPLACE THE EXPERTISE OF AN EXPERIENCED CRYSTALLOGRAPHIC REFEREE.

No syntax errors found. CIF dictionary Interpreting this report

Datablock: 1

```
Bond precision:  C-C = 0.0030 A          Wavelength=0.71073

Cell:              a=11.651(2)      b=13.671(3)      c=35.540(6)
                   alpha=90         beta=90          gamma=90
Temperature:       100 K

                Calculated                 Reported
Volume          5660.8(18)                 5660.5(18)
Space group     P b c a                    P b c a
Hall group      -P 2ac 2ab                 -P 2ac 2ab
Moiety formula  C16 H18 N2 O2              C16 H18 N2 O2
Sum formula     C16 H18 N2 O2              C16 H18 N2 O2
Mr              270.32                     270.32
Dx,g cm-3       1.269                      1.269
Z               16                         16
Mu (mm-1)       0.085                      0.085
F000            2304.0                     2304.0
F000'           2304.97
h,k,lmax        14,16,42                   14,16,42
Nref            5181                       5168
Tmin,Tmax       0.966,0.993                0.683,0.745
Tmin'           0.948

Correction method= # Reported T Limits: Tmin=0.683 Tmax=0.745
AbsCorr = MULTI-SCAN

Data completeness= 0.997          Theta(max)= 25.339

R(reflections)= 0.0410( 3741)     wR2(reflections)= 0.1044( 5168)

S = 1.029                   Npar= 375
```

The following ALERTS were generated. Each ALERT has the format
test-name_ALERT_alert-type_alert-level.
Click on the hyperlinks for more details of the test.

Alert level G

```
PLAT002_ALERT_2_G Number of Distance or Angle Restraints on AtSite          4 Note
PLAT063_ALERT_4_G Crystal Size Possibly too Large for Beam Size ..       0.63 mm
PLAT172_ALERT_4_G The CIF-Embedded .res File Contains DFIX Records          1 Report
PLAT860_ALERT_3_G Number of Least-Squares Restraints .............          2 Note
PLAT910_ALERT_3_G Missing # of FCF Reflection(s) Below Theta(Min).          1 Note
PLAT912_ALERT_4_G Missing # of FCF Reflections Above STh/L=  0.600         11 Note
PLAT978_ALERT_2_G Number C-C Bonds with Positive Residual Density.          6 Info
```

```
   0 ALERT level A = Most likely a serious problem - resolve or explain
   0 ALERT level B = A potentially serious problem, consider carefully
   0 ALERT level C = Check. Ensure it is not caused by an omission or oversight
   7 ALERT level G = General information/check it is not something unexpected

   0 ALERT type 1 CIF construction/syntax error, inconsistent or missing data
   2 ALERT type 2 Indicator that the structure model may be wrong or deficient
   2 ALERT type 3 Indicator that the structure quality may be low
   3 ALERT type 4 Improvement, methodology, query or suggestion
   0 ALERT type 5 Informative message, check
```

It is advisable to attempt to resolve as many as possible of the alerts in all categories. Often the minor alerts point to easily fixed oversights, errors and omissions in your CIF or refinement strategy, so attention to these fine details can be worthwhile. In order to resolve some of the more serious problems it may be necessary to carry out additional measurements or structure refinements. However, the purpose of your study may justify the reported deviations and the more serious of these should normally be commented upon in the discussion or experimental section of a paper or in the "special_details" fields of the CIF. checkCIF was carefully designed to identify outliers and unusual parameters, but every test has its limitations and alerts that are not important in a particular case may appear. Conversely, the absence of alerts does not guarantee there are no aspects of the results needing attention. It is up to the individual to critically assess their own results and, if necessary, seek expert advice.

Publication of your CIF in IUCr journals

A basic structural check has been run on your CIF. These basic checks will be run on all CIFs submitted for publication in IUCr journals (*Acta Crystallographica*, *Journal of Applied Crystallography*, *Journal of Synchrotron Radiation*); however, if you intend to submit to *Acta Crystallographica Section C* or *E* or *IUCrData*, you should make sure that full publication checks are run on the final version of your CIF prior to submission.

Publication of your CIF in other journals

Please refer to the *Notes for Authors* of the relevant journal for any special instructions relating to CIF submission.

PLATON version of 16/05/2021; check.def file version of 13/05/2021

Datablock 1 - ellipsoid plot

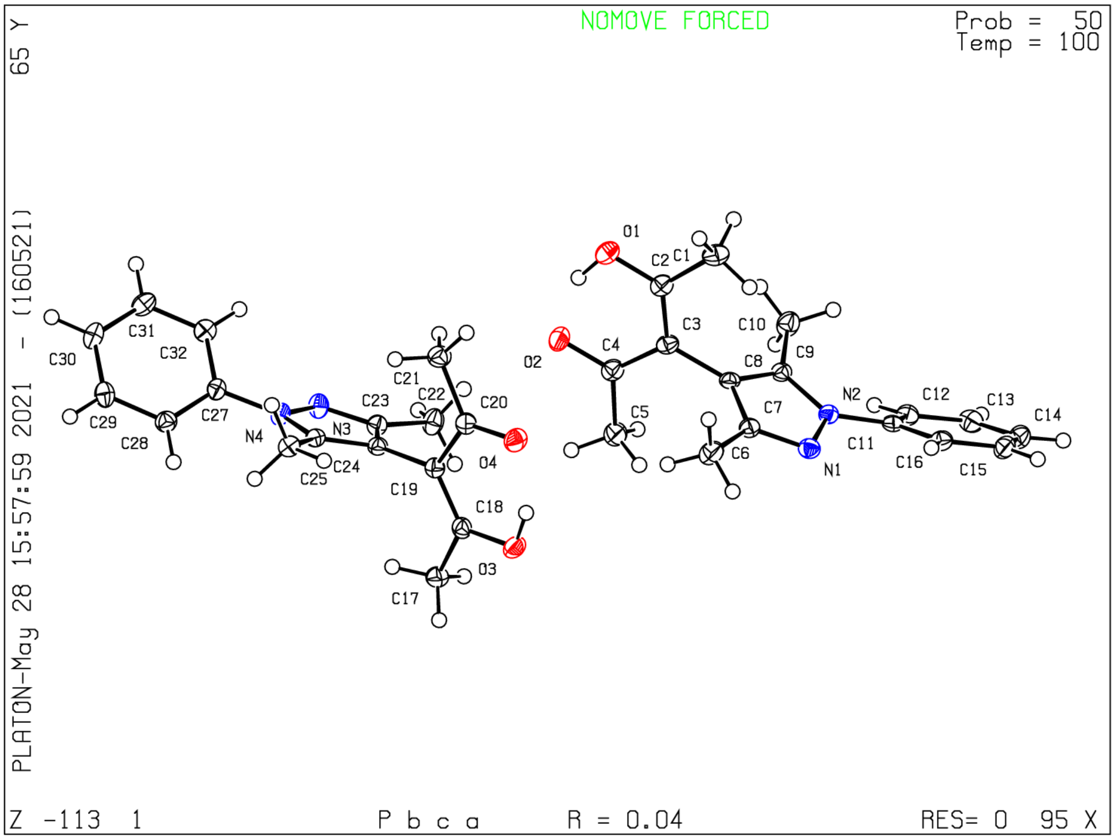

Supplement: Supplementary file 1 [file molecules-26-03986-s001.zip › suppinfo/1_checkcif.pdf]
