# Supplementary material for: Staring at the Naked Goddess: Unraveling the Structure and Reactivity of Artemis Endonuclease Interacting with a DNA Double Strand
Source: Molecules. 2021 Jun 29;26(13):3986. doi: 10.3390/molecules26133986 (PMC8271620; doi:10.3390/molecules26133986)

checkCIF/PLATON report

Structure factors have been supplied for datablock(s) 2

THIS REPORT IS FOR GUIDANCE ONLY. IF USED AS PART OF A REVIEW PROCEDURE FOR PUBLICATION, IT SHOULD NOT REPLACE THE EXPERTISE OF AN EXPERIENCED CRYSTALLOGRAPHIC REFEREE.

No syntax errors found. CIF dictionary Interpreting this report

Datablock: 2

Bond precision:	C-C = 0.0032 A		Wavelength=0.71073
Cell:	a=10.963(3)	b=10.240(3)	c=18.038(5)
	alpha=90	beta=93.269(6)	gamma=90
Temperature:	100 K		

	Calculated	Reported
Volume	2021.7(10)	2021.8(10)
Space group	P 21/c	P 21/c
Hall group	-P 2ybc	-P 2ybc
Moiety formula	C16 H19 N2 O2, 0.5(C6 F4 I2), Cl	C16 H19 N2 O2, 0.5(C6 F4 I2), Cl
Sum formula	C19 H19 Cl F2 I N2 O2	C19 H19 Cl F2 I N2 O2
Mr	507.71	507.71
Dx,g cm-3	1.668	1.668
Z	4	4
Mu (mm-1)	1.750	1.750
F000	1004.0	1004.0
F000’	1003.14	
h,k,lmax	18,17,30	18,16,29
Nref	9744	9541
Tmin,Tmax	0.722,0.811	0.624,0.747
Tmin’	0.698	

Correction method= # Reported T Limits: Tmin=0.624 Tmax=0.747
AbsCorr = MULTI-SCAN

Data completeness= 0.979 Theta(max)= 36.248

R(reflections)= 0.0406(6927) wR2(reflections)= 0.0871(9541)

S = 1.041 Npar= 254

The following ALERTS were generated. Each ALERT has the format
test-name_ALERT_alert-type_alert-level.
Click on the hyperlinks for more details of the test.

Alert level C

```
RINTA01_ALERT_3_C  The value of Rint is greater than 0.12
            Rint given   0.127
```

Alert level G

```
PLAT002_ALERT_2_G Number of Distance or Angle Restraints on AtSite          3 Note
PLAT020_ALERT_3_G The Value of Rint is Greater Than 0.12 .........      0.127 Report
PLAT176_ALERT_4_G The CIF-Embedded .res File Contains SADI Records          1 Report
PLAT303_ALERT_2_G Full Occupancy Atom H1        with # Connections       2.00 Check
```

Author Response: In contrast to the free ligand, no localization of the enol H could be observed. Therefore, the enol H was placed in the middle of both oxygen atoms of the acetylacetone moiety with a distance restraint.

```
PLAT434_ALERT_2_G Short Inter HL..HL Contact I1       ..Cl1             3.17 Ang.
                                                      x,y,z  =      1_555 Check
PLAT860_ALERT_3_G Number of Least-Squares Restraints .............          1 Note
PLAT912_ALERT_4_G Missing # of FCF Reflections Above STh/L=  0.600        198 Note
PLAT978_ALERT_2_G Number C-C Bonds with Positive Residual Density.          8 Info
```

---

0 **ALERT level A** = Most likely a serious problem - resolve or explain
0 **ALERT level B** = A potentially serious problem, consider carefully
1 **ALERT level C** = Check. Ensure it is not caused by an omission or oversight
8 **ALERT level G** = General information/check it is not something unexpected

0 ALERT type 1 CIF construction/syntax error, inconsistent or missing data
4 ALERT type 2 Indicator that the structure model may be wrong or deficient
3 ALERT type 3 Indicator that the structure quality may be low
2 ALERT type 4 Improvement, methodology, query or suggestion
0 ALERT type 5 Informative message, check

It is advisable to attempt to resolve as many as possible of the alerts in all categories. Often the minor alerts point to easily fixed oversights, errors and omissions in your CIF or refinement strategy, so attention to these fine details can be worthwhile. In order to resolve some of the more serious problems it may be necessary to carry out additional measurements or structure refinements. However, the purpose of your study may justify the reported deviations and the more serious of these should normally be commented upon in the discussion or experimental section of a paper or in the "special_details" fields of the CIF. checkCIF was carefully designed to identify outliers and unusual parameters, but every test has its limitations and alerts that are not important in a particular case may appear. Conversely, the absence of alerts does not guarantee there are no aspects of the results needing attention. It is up to the individual to critically assess their own results and, if necessary, seek expert advice.

Publication of your CIF in IUCr journals

A basic structural check has been run on your CIF. These basic checks will be run on all CIFs submitted for publication in IUCr journals (*Acta Crystallographica*, *Journal of Applied Crystallography*, *Journal of Synchrotron Radiation*); however, if you intend to submit to *Acta Crystallographica Section C* or *E* or *IUCrData*, you should make sure that full publication checks are run on the final version of your CIF prior to submission.

Publication of your CIF in other journals

Please refer to the *Notes for Authors* of the relevant journal for any special instructions relating to CIF submission.

PLATON version of 16/05/2021; check.def file version of 13/05/2021

Datablock 2 - ellipsoid plot

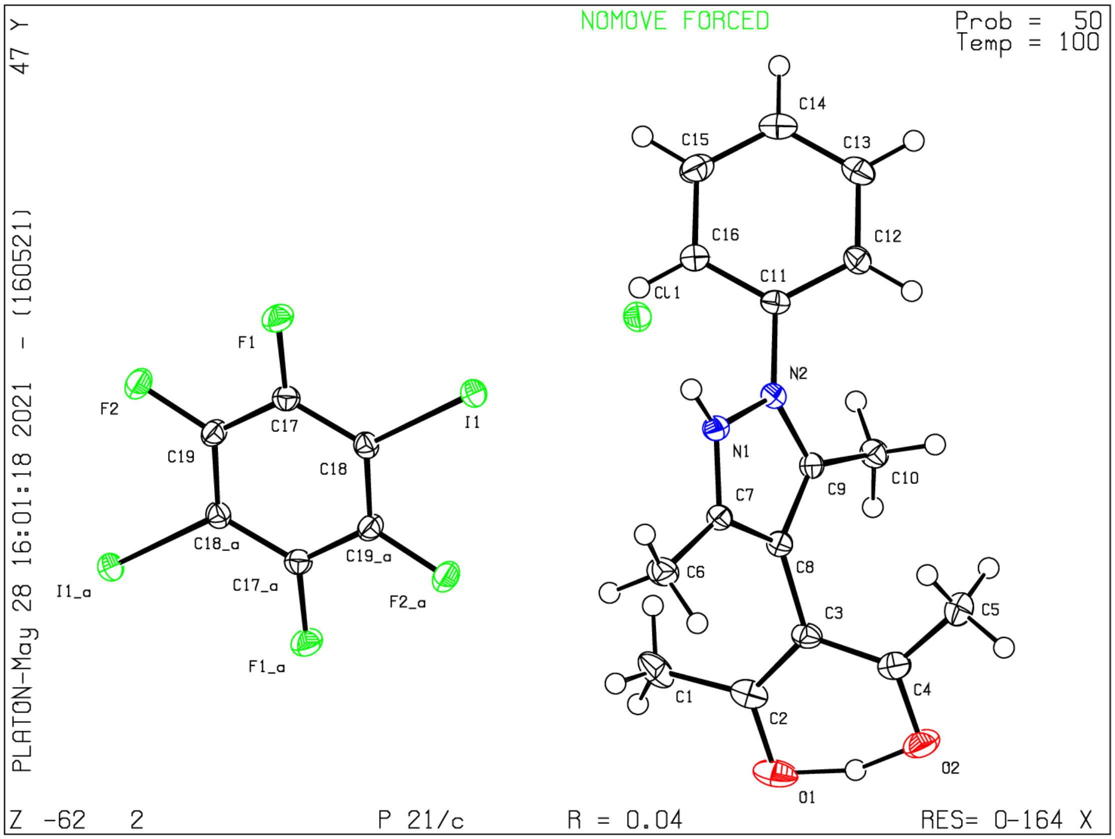

Supplement: Supplementary file 1 [file molecules-26-03986-s001.zip › suppinfo/2_checkcif.pdf]
