# Supplementary material for: Staring at the Naked Goddess: Unraveling the Structure and Reactivity of Artemis Endonuclease Interacting with a DNA Double Strand
Source: Molecules. 2021 Jun 29;26(13):3986. doi: 10.3390/molecules26133986 (PMC8271620; doi:10.3390/molecules26133986)
Supplement: Supplementary file 1 [file molecules-26-03986-s001.zip › suppinfo/SI_revised.pdf]

Supplementary Materials: Hydrogen-Bonded and Halogen-Bonded: Orthogonal Interactions for the Chloride Anion of a Pyrazolium Salt

Steven van Terwingen[1], Daniel Brüx[1], Ruimin Wang[1] and Ulli Englert[1,2,*]

1. Powder Diffractograms

Simulated powder patterns refer to the temperature of the single-crystal measurement (100 K), corresponding to a smaller unit cell and thus to larger 2θ in reciprocal space. This results in a slight systematic shift between experimental and simulated diffraction patterns.

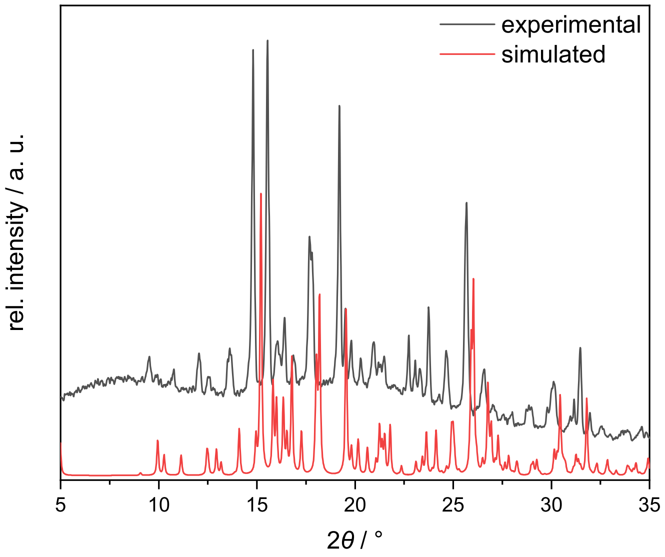

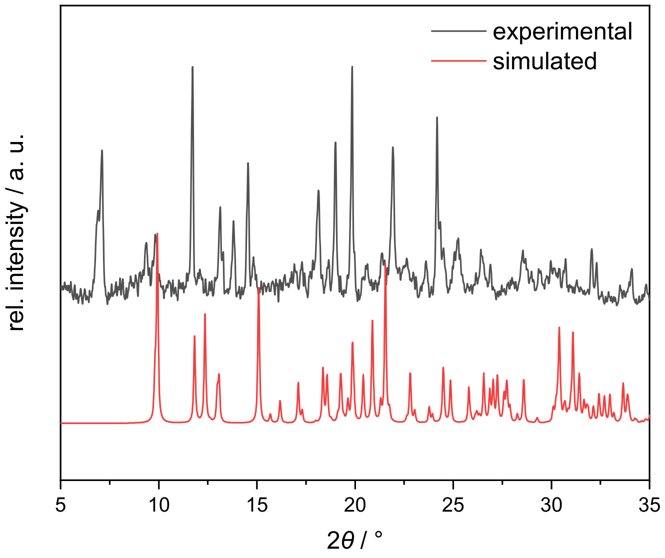

Figure S1. Simulated and experimental powder patterns of **1** (top) and **2** (bottom).

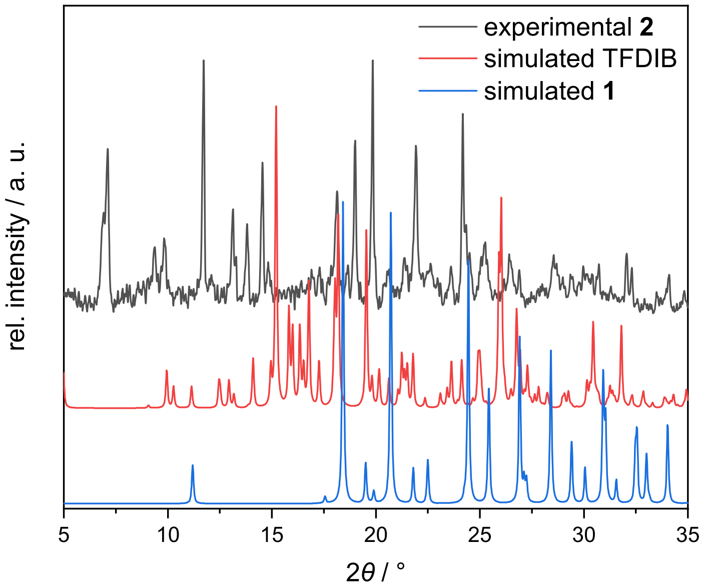

Figure S2. Experimental powder patterns of **2** and simulated patterns of **1** and TFDIB [1].

2. Crystal Data and Refinement Results

Table S1: Crystal data and refinement results for SCXRD data for **1** and **2** measured at $T = 100\,\mathrm{K}$.

Compound	**1**	**2**
Moiety formula	$C_{16}H_{18}N_2O_2$	$C_{16}H_{19}N_2O_2$, 0.5($C_6F_4I_2$), Cl
Formula weight / $\mathrm{g\,mol^{-1}}$	270.32	507.71
Crystal habit	colorless plate	colorless block
Crystal size / $\mathrm{mm^3}$	$0.63 \times 0.34 \times 0.08$	$0.20 \times 0.16 \times 0.12$
Crystal system	orthorhombic	monoclinic
Space group (*No.*)	*Pbca* (*61*)	$P2_1/c$ (*14*)
a / Å	11.651(2)	10.963(3)
b / Å	13.671(3)	10.240(3)
c / Å	35.540(6)	18.038(5)
β / °	90	93.269(6)
V / $\mathrm{Å^3}$	5660.5(18)	2021.8(10)
Z	16	4
D_{calc} / $\mathrm{g\,cm^{-3}}$	1.269	1.668
μ / $\mathrm{mm^{-1}}$	0.085	1.750
$\sin(\theta_{max})/\lambda$ / $\mathrm{Å^{-1}}$	0.60	0.83
total/unique refl.	45107/5168	114651/9541
observed refl.	3741	6927
No. of parameters	375	254
R_{int}	0.0953	0.1275
$R_1(I > 2\sigma(I))$	0.0410	0.0406
wR_2 (all data)	0.1044	0.0871
S (all data)	1.031	1.041
ρ_{min}/ρ_{max} / $e\,\mathrm{Å^{-3}}$	−0.257/0.265	−0.675/0.765
CCDC #	2086575	2086574

3. NMR Spectra

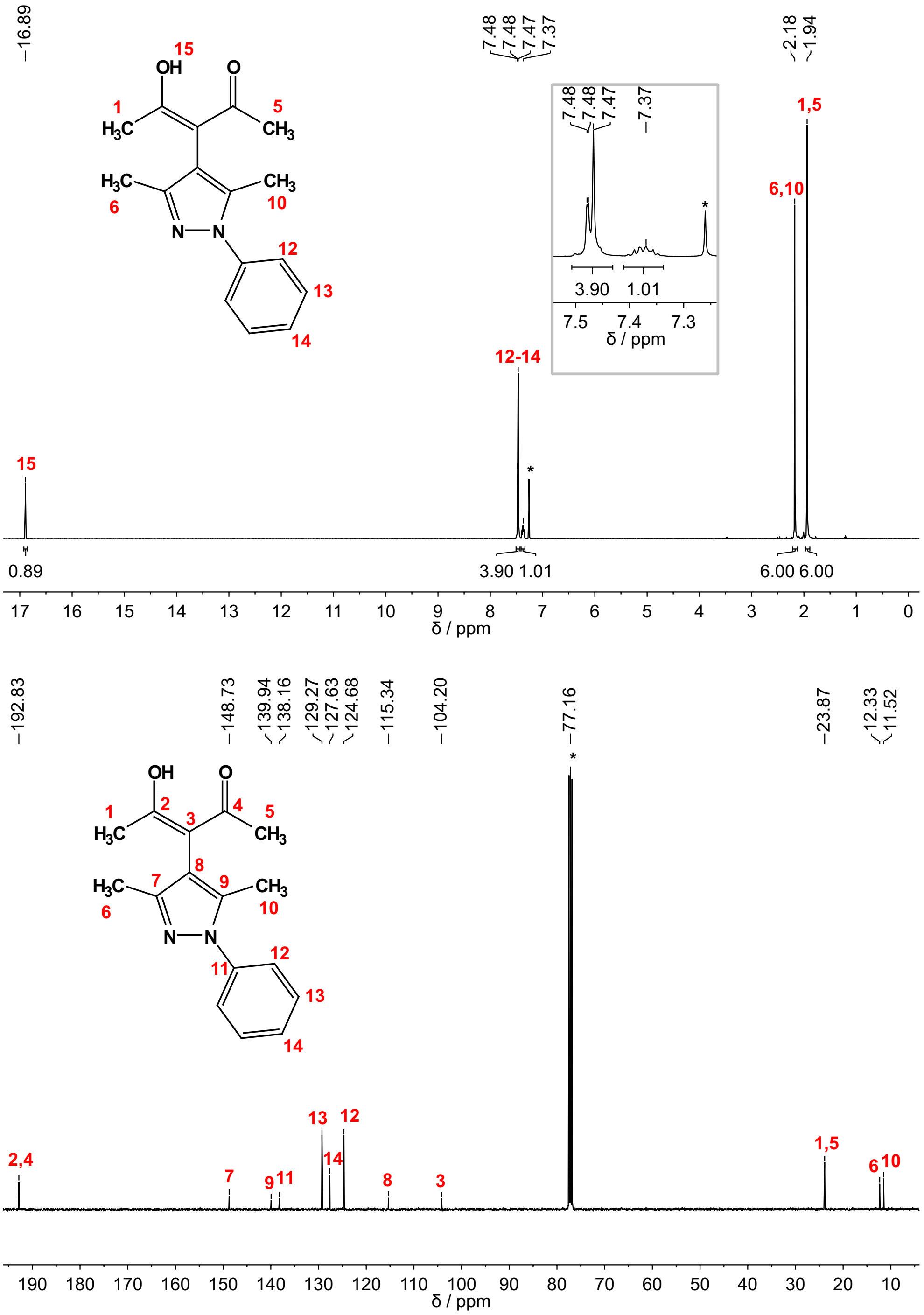

Figure S3. ^{1}H (top) and $^{13}C\{^{1}H\}$ (bottom) NMR spectra of **1** measured in $CDCl_3$ (*) at room temperature.

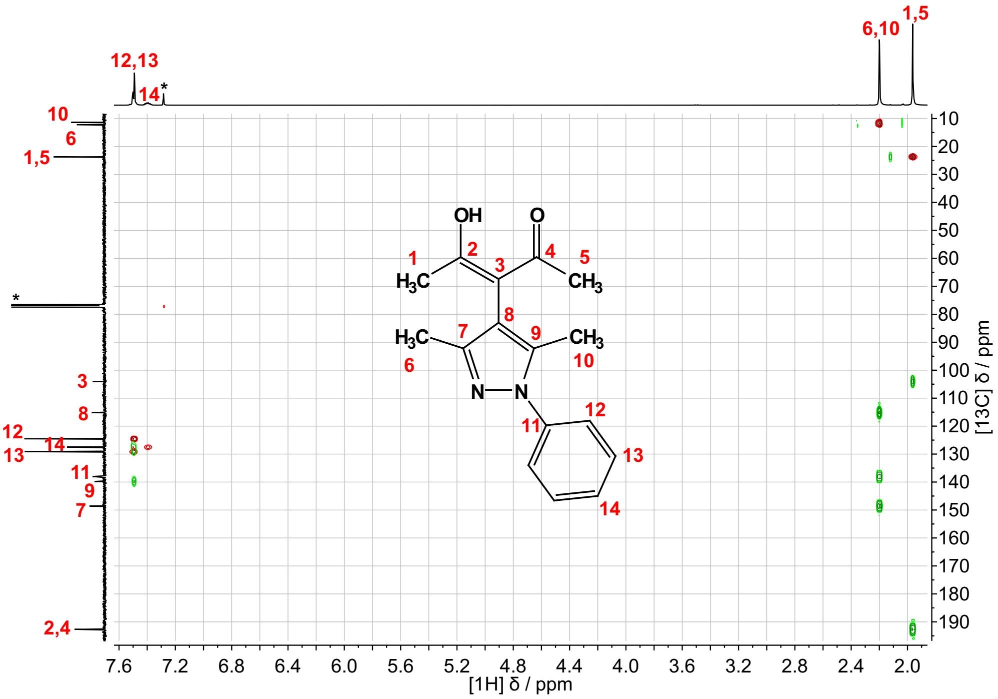

Figure S4. HSQC (red) and HMBC (green) NMR spectra of **1** measured in $CDCl_3$ (*) at room temperature.

4. Computational Details

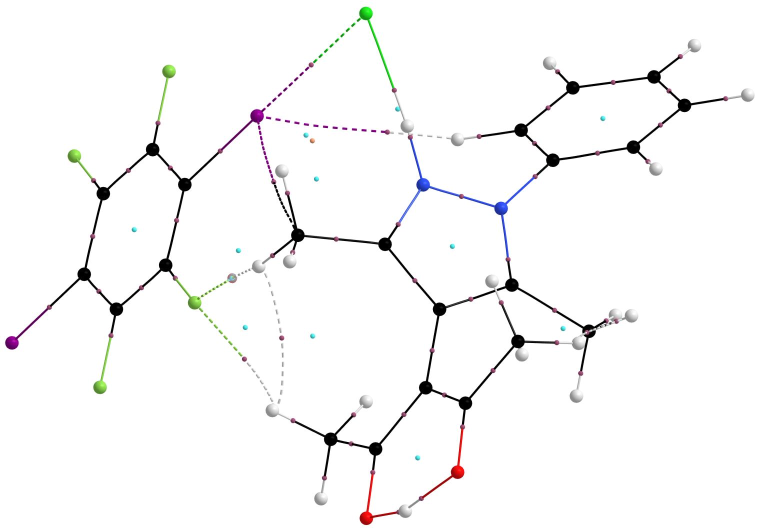

Figure S5. Structure fragment used for the single point calculation discussed in the main text, section 2.2.

Table S2: Topological properties of interactions at their bond critical point $(3, -1)$ of **2**.

Bond	ρ / e Å^{-3}	$\nabla^2\rho$ / e Å^{-5}	bond path / Å	G / a. u.	G/ρ / a. u.	V / a. u.	E / a. u.
I1···Cl1	0.129	1.184	3.1654	0.0110	0.58	–0.0097	0.0123
Cl1···H1N	0.321	1.785	2.0680	0.0300	0.63	–0.0415	–0.0115
I1–C1	0.808	1.403	2.0902	0.0740	0.62	–0.1333	–0.0594
N1–H1N	2.043	–54.371	1.0109	0.0471	0.16	–0.6582	–0.6111
C17–C18	2.066	–23.823	1.3892	0.1106	0.36	–0.4683	–0.3577
N1–N2	2.477	–23.328	1.3603	0.1790	0.49	–0.5999	–0.4209
N1–C7	2.051	4.135	1.3359	0.3981	1.31	–0.7532	–0.3552
N2–C9	1.642	–2.498	1.4352	0.2310	0.95	–0.4879	–0.2569
F1–C17	1.672	6.365	1.3501	0.3505	1.41	–0.6349	–0.2844
O1–H1	1.957	–56.928	0.9975	0.0740	0.26	–0.7385	–0.6645
O2···H1	0.607	3.338	1.5687	0.0662	0.74	–0.0977	–0.0315
O1–C2	2.099	8.129	1.2970	0.4452	1.43	–0.8061	–0.3609
O2–C4	2.207	5.954	1.2842	0.4522	1.38	–0.8426	–0.3904
C3–C8	1.706	–17.040	1.4790	0.0736	0.29	–0.3240	–0.2504
C9–C10	1.685	–16.762	1.4863	0.0741	0.30	–0.3220	–0.2479
C3–C4	1.964	–22.066	1.4181	0.0964	0.33	–0.4216	–0.3253
C13–C14	2.067	–24.967	1.3895	0.1006	0.33	–0.4602	–0.3596

References

1. Chaplot, S.L.; McIntyre, G.J.; Mierzejewski, A.; Pawley, G.S. The High-Temperature Phase of 1,2,4,5-Tetrafluoro-3,6-diiodobenzene and the Phase Transition. *Acta Crystallogr.* **1981**, *B37*, 2210–2214. doi:10.1107/S0567740881008406.
